# Iranian mothers’ perceptions of contextual factors helping them manage pain in labor

**DOI:** 10.1186/s12884-024-07012-x

**Published:** 2024-12-04

**Authors:** Faeghe Deljoo Ghamgosar, Muhammad Sadra, Hassan Yari, Mina Honarbakhsh

**Affiliations:** 1 Present Address: Iran University of Science and Technology, Tehran, Iran; 2 University of Zanjan, Zanjan, Iran; 3 Kidney Transplantation, Department of Surgery, School of Medicine, Ayatollah Mousavi Hospital, Zanjan, Iran; 4 Obstetrics & Gynecology, Ayatollah Mousavi Hospital, Zanjan, Iran

**Keywords:** Labor pain, Pain management, Contextual factors, Childbirth, Caesarean section, Phenomenology

## Abstract

**Background:**

The rate of caesarean section in Iran has significantly increased. The main reason cited by Iranian mothers for it is labor pain and fear of it. However, the experience of pain during childbirth has different meanings for mothers in various conditions. Therefore, this study aims to examine the components from the mothers’ perspective that helped them manage pain in labor. The findings of this article may help prevent unnecessary medical interventions.

**Methods:**

The current article is one component of a mixed-method study conducted in Iran. Its primary objective was to develop protocols for maternity unit design to enhance maternal satisfaction, promote husbands’ involvement, and improve pain management. The current study focuses on examining contextual components that are effective in managing pain among 25 mothers selected through stratified purposive sampling. Data collection from mothers who gave birth in four large hospitals in Iran was carried out through unstructured in-depth interviews. The data were analyzed using interpretative phenomenological analysis.

**Findings:**

The data show that two major environmental factors affect mothers’ perception of pain and their ability to control it: (1) Internal and (2) External environments. The main components of the internal factor are identified as “Personal beliefs”, “Self-confidence and lack of fear”, and the external environment includes three subfactors: “Spatial environment”, “Social environment”, and “Interactive environment”. The overall meaning attributed to pain caused by these factors determines how mothers can cope with it.

**Conclusion:**

In this study, self-confidence and a positive mindset were significant factors in managing pain during labor among this group of mothers. Furthermore, each mother’s perception of pain was shaped by a combination of physical, social, and interactive influences. The research identified the importance of support during labor according to mothers’ individual, subjective needs, while improving the physical environment, with a view to reducing interventions and promoting positive experiences for mothers and husbands. Consequently, enhancing internal resources and the birthing environment during labor which involve identifying the optimal blend of physical, mental, and emotional strategies tailored to the specific needs of mothers, can be considered to a potential reduction in the perceived intensity of pain in future.

## Introduction

The caesarean section rate in Iran is 48%, with some cities exceeding 66% [1]. Iran ranks first globally [2] and in Asia [3] for this rate. However, the World Health Organization (WHO) indicates that a national caesarean section rate above 10% does not improve maternal and neonatal outcomes [4]. The exact rate of caesarean sections on maternal demand (CBMD) in Iran is unclear [5]. Given the higher complication rates for mothers and fetuses, significant healthcare costs [6], and increased mortality risk [7], reducing unnecessary caesarean sections is crucial. Factors contributing to its prevalence in Iran include personal beliefs [8], social influences, fear of vaginal birth [1, 8], and physician recommendations [1]. Notably, fear of vaginal birth is a primary reason primiparous women opt for caesarean delivery [1, 9]. Additionally, fear and anxiety can lead to negative birth experiences [10] and disrupt the neurohormonal processes essential for labor, increasing the need for interventions [11].

The majority of mothers undergo pain during labor, with many experiencing it as the most intense pain they have ever endured in their lives. Although labor pain is linked to the same fundamental physiological process, it is noteworthy that not all mothers experience it in the same manner. Mothers’ evaluations of labor pain can vary widely, ranging from excruciating to even pleasurable, depending on the individual or the specific occasion [12]. While some mothers can effectively cope with the pain, requiring minimal assistance and reporting positive labor experience, others may have a negative perception of pain and seek medical intervention to prevent or alleviate it [13, 14].

Labor pain is thus a complex, unique [12], and multidimensional [15] experience. Mothers vary in their ability to cope with it due to their individual lived experiences. Nevertheless, the intricate nature of this subjective phenomenon makes it challenging to manage [12]. Although many pharmacological and nonpharmacological methods have been adopted to support mothers during labor, the rising rate of caesarean sections suggests that the existing methods of support may not be adequate [11]. While various pain management strategies are available, including environmental and social approaches, pharmacological interventions are frequently employed [12, 16]. In Iran, the administration of oxytocin during labor is considered a standard procedure [17, 18], even though these interventions are not associated with positive labor experiences in women [19, 20]. Furthermore, they can contribute to a decrease in the rates of vaginal birth [16, 21] and increase the likelihood of newborns being admitted to the NICU (Neonatal Intensive Care Unit) [22].

Consequently, it is crucial to explore environmental and contextual approaches as components of hospital pain relief strategies because by doing so, significant benefits will be provided to both mothers and their babies without causing additional harm [23, 24]. However, research on this subject has been limited thus far, and some researchers have recommended conducting qualitative research to identify cultural factors related to labor pain management [25] or harness mothers’ inherent capabilities and leverage the social environment surrounding them to reduce their reliance on pain interventions [12].

Considering that cultural, economic, and social differences in each community can influence human experiences [26], this study was conducted with a group of Iranian women to examine the effect of contextual factors on pain management. Analyzing environmental pain relief strategies can assist caregivers and healthcare designers in gaining a better understanding of maternal needs, ultimately enabling them to provide woman-centered care [27]. Based on the literature, this research represents one of the few qualitative studies, not only in Iran but also in developed countries that survey mothers’ experiences of labor pain and the environmental factors affecting it.

## Method

### Study design

The Ethics Committee of Iran University of Science and Technology, Tehran, Iran approved the study protocol (Code: 2625391). This study is part of a mixed-methods study aiming to develop protocols for designing the maternity department. The focus of this research includes aspects such as mother’s satisfaction, husband’s presence, and pain management and labor progress. The objective of this study was to gain insight into the perspective of a group of Iranian mothers regarding factors they perceive either as helpful or traumatizing for their labor pain. According to modern pain science, pain is a subjective and multidimensional [15] phenomenon that, in principle, cannot be observed or objectively assessed [28] and is perceived within a personal context [29, 30].

To evaluate the impact of contextual strategies on labor pain, unstructured in-depth interviews were conducted to incorporate pain into the assessment. Interpretative phenomenological analysis (IPA) was employed to analyze these interviews, making the data comprehensible for the readers. IPA developed by Jonathan Smith, is designed to enable a rigorous examination of subjective experiences and social cognition [31]. As labor pain can only be accessed through the conscious mind, phenomenology was an appropriate approach. It serves as both a philosophical and a theoretical framework based on the notion that certain phenomena can only be truly understood from the perspective of the person experiencing them [32, 33]. The IPA method aims to examine lived experiences in their contexts while acknowledging the active role of the researcher in the process [34].

### Setting

The research settings included tertiary educational hospitals and private and organizational hospitals located in the cities of Tehran and Zanjan. Tehran is the capital of Iran, while Zanjan is a metropolitan city situated in the northwestern region of the country. The participants in this study were primiparous mothers who had given birth to a healthy baby and did not have any mental disorders, such as depression.

### Sampling and participants

The sampling method used in this study was stratified purposive sampling. It aimed to represent mothers at both higher and lower risk of complications, as well as different models of care, including midwifery-led care with or without continuous care from a known midwife and with or without support from family members. The participants in this study were primiparous mothers who gave birth to full-term babies. Prior to childbirth, they were not scheduled for a planned caesarean section and were expecting a vaginal birth. However, depending on the progress or lack of progress of labor, their mode of delivery changed and included physiological, natural with medical interventions, or caesarean delivery.

### Data collection

All participants provided informed written consent and were assured that their identity and information would be kept confidential throughout all stages of research, including implementation and publication. Then, the qualitative data were collected through unstructured in-depth interviews using open-ended questions such as “What factors attracted your attention to, or diverted your attention from, the birth?”, “Could you please describe the pain you experienced from the onset of first stage of labor until the birth? “, and “Tell me about the impact of your surroundings and the people present around you”. When necessary, prompts were provided to encourage women to explore and describe their pain experience from sensory, affective and cognitive perspectives. FDG, first author, conducted interviews in locations where mothers felt at ease (their own homes, medical centers, and universities). The duration of each interview was between 40 and 60 min. The interviews were conducted within a span of one year and six months from January 2020 to June 2021 within 2 to 4 weeks after giving birth to minimize any false positive or negative effects resulting from the excitement of having a baby or the possible fading of the pain experience [25].

### Data analysis

The analysis was carried out according to IPA [35]. The reason for using IPA as an analytical method was to create a better understanding of the experience of the mothers of pain and contextual effect on labor progress. No software application was used, and the handwritten method was applied for data analysis. This research was seen as a dynamic process where analysis involved continuous interpretation by the researcher. This approach was linked to a two-stage or double hermeneutic process [36]. The analysis was carried out in several steps, outlined as follows:

1. Interviews were transcribed verbatim, and to ensure anonymity, participant names were not used within the quotes.
2. The first author (FDG) began by examining the transcript of one interview in detail before moving on to the other interviews. Each interview was then read through several times to gain a first understanding. Then, the meaning units were identified and organized into categories based on their differences or similarities by first author. The corresponding author (MS), an expert in qualitative and contextual methods, supervised this. It was then confirmed by HY and MH (third and fourth authors).
3. The connections between meaning units were analyzed, with some units grouped together. Key phrases that captured the core of the informant’s statements, like “My husband would tell me that we had been together from the beginning and would stay together to the end,” were used to identify related topics, such as “Social Environment.“.
4. Organizing the analysis to enhance comprehension of the processes and structures revealed by the research data.
5. Refining the themes further by creating a summary to distinguish between main themes and subthemes.

Direct quotes from the interviews are included in the text as quotations. The findings were organized into two main themes: Internal and External Environments, along with five sub-themes: Self-Confidence and Lack of Fear, Personal Beliefs, Spatial Environment, Social Environment, and Interactive Environment. A summary of these is shown in Table 1. Examples of the analysis in the second theme “External Environment” are shown in Table 2.

**Table 1:** Summary of findings on mothers’ perception of the Context’s impact on Pain

Themes	Internal Environment	External Environment
**Sub-themes**	Self-Confidence and Lack of Fear	Spatial Environment
	Personal Beliefs	Social Environment
		Interactive Environment

**Table 2:** Example of structural analysis of the second topic: “External Environment”

Meaning Unit	Connecting the Meaning Unit into Themes	Sub- themes	Main Themes
“When I entered the labor ward, I was in pain. I had thought the ward was filled with mothers screaming, but it was quiet… There were no hospital gowns or white walls in sight… They even allowed me to wear the clothes I wanted… I still had a lot of pain, but I no longer felt negative… I felt like I was a guest at someone’s, and believed I could handle the pain.” “I was in pain and expected it to get worse. However, the birthing ball and a warm shower helped me a lot.”	Providing a sense of security, enhancing capability, and addressing negative assumptions can shape the impact of the physical environment on perception of pain.	Spatial Environment	External Environment
“I was happy that my husband was by my side. He knew nothing about childbirth, and my pain did not lessen because of him. Nevertheless, his presence was reassuring. He would tell me that we had been together from the beginning and would stay together to the end.” “The nurses were very calm. When I complained about my pain and stress for the umpteenth time, they smiled and reassured me that everything was under control. They never treated me disrespectfully at all.”	Support, reassurance, respect, and effective management strategies are related to the childbirth experience.	Social Environment	
“My husband was present throughout labor. However, during examinations, since all the medical staff were women, he felt embarrassed. He did not have a proper physical placement in the room. I was more concerned about him rather than focusing on my labor.”	Communication with both the place and the people around creates a sense of belonging and control.	Interactive Environment	

### Reliability and rigor

This study established its reliability using various methodological approaches. “Credibility” [37–39] by Member Checking and Peer Debriefing [40], “transparency” [37], “dependability” [38, 39], and “conformability” [38, 41] were assessed to confirm the accuracy of study results. To ensure rigor in the analysis, interpretative phenomenological analysis (IPA) was applied, with multiple researchers involved in the coding process to cross-check interpretations and reduce bias. The data were also provided to the second (MS) and third author (HY) to confirm the consistency of the categories with the mothers’ statements. The coded texts were provided to some mothers for checking the accuracy of the interpretations [40]. As subjective nature of interpretative phenomenology may inherently challenge the notion of objectivity, all the authors considered an ongoing dialogue about the balance between their interpretation and mothers voice. The study also employed “reflexivity” [42], with researchers reflecting on their roles and potential biases. Additionally, data saturation was reached, further enhancing the reliability of the findings. Also, the Standards for Reporting Qualitative Research Checklist (SRQR) was used to write this article [43]. Previous publications include more details on the rigor of the study [44, 45].

## Findings

### Information on mothers

Although data saturation was achieved after conducting 18 interviews, a total of 25 mothers, ranging in age from 19 to 40, were interviewed. This included 12 mothers with high-risk pregnancies and 13 mothers with low-risk pregnancies. Table 3 depicts participant characteristics. Table 4 provides additional essential descriptors of women’s pregnancies and births, indicated in superscript at the end of quotes. These include pregnancy risk levels, onset and progress of labor, and mode of birth.

**Table 3:** Mothers’ characteristics

Variables	***n*** = 25
**Age**	
19 to 25	8
26 to 32	10
33 to 40	7
**Education**	
High School	14
Tertiary	11
**Onset of labor**	
Spontaneous	8
Augmented	6
Induced	11
**Birth outcomes**	
Physiological vaginal birth (unassisted)	5
Normal vaginal birth (epidural/spinal)	13
Unplanned caesarean section	7
**Pregnancy risk level**	
High	12
Low	13

**Table 4:** Mother descriptors

Pregnancy risk level	Onset of labor	Mode of delivery
High = H	Spontaneous = SP	Physiological vaginal birth = PVD
Low = L	Augmented = AU	Normal vaginal birth = NVD
	Induced = IN	Caesarean section = CS

### Mothers’ perception of the impact of the environment on pain

The descriptions provided by mothers regarding labor pain and the contextual factors affecting it, as observed in this study, demonstrated the intricate nature of this relationship. The overall theme emerging from the data is that two major environmental factors affected this group mothers’ perception of pain and their ability to control it: (1) internal environment and (2) external environment. The data indicate that mothers affected by their internal world had a positive or negative interpretation of labor pain. It was the meaning of the pain that shaped the pain experience and their ability to respond to it. On the other hand, although the mother experienced everything through her mentality, her internal image and interpretation of the external world shaped the emotional and cognitive values that gave meaning to pain. Therefore, the mother’s perception of context and external phenomena was also directly linked to her ability to control pain. The ultimate meaning attributed to the pain determined how she could cope with it.

#### Internal environment

In this study, the personal characteristics of mothers, which were the result of their life experience within Iranian society, were referred to as the “internal environment”. According to the opinions of the participants, the main components of this factor were identified as “personal beliefs” and “self-confidence and lack of fear”. Mothers, under the influence of these two components, were divided into two categories in terms of their pain management abilities.

The first group of mothers, who perceived pain as a necessary and natural aspect of childbirth, possessed the ability to cope with pain and did not seek medical interventions. In this study, it was observed that some mothers, influenced by the cultural conditions of Iran, endured labor pain while adhering to their religious beliefs. These mothers perceived labor pain as a natural and inevitable part of the process of becoming a mother. Employing concepts such as “reliance on God” and considering the mental aspect of pain effectively demonstrated how a mother’s cultural and religious attitude could affect pain management.

*I understand that these pains are natural*,* and I believe that God has ordained them for mothers. I see it as a test that every mother must endure to achieve her goal. Since no one has beaten me or caused me any harm*,* I can manage it.*^*H, IN, NVD*^.

*I always place my trust in God when facing challenges. It is a good thing (childbirth)*,* and I am confident that God will not leave me alone.*^*L, SP, PVD*^.

*There is pain*,* but not to the extent that it cannot be endured. God has created the body in a way that we can overcome pain.*^*H, IN, NVD*^.

Some others had a positive conception of pain and displayed self-confidence in managing it. They had participated in childbirth preparation classes. Thanks to the knowledge and personal empowerment they gained from these classes, they were well aware of each stage of labor, experiencing no surprises along the way.

… *When the intervals between my contractions became shorter*,* I felt more pain. I would take deep breaths to control the pain*,* something I used to do when getting injections.*^*L, IN, NVD*^.

*There was pain*,* but it was bearable because everything was going according to my plan. I was not afraid. In contrast*,* I was happy because I was getting closer to holding my daughter in my arms.*^*H, AU, NVD*^.

*First*,* my bag of waters [amniotic sac] broke. I realized it had begun… contractions started. The more pain I felt*,* the more I knew I was getting closer to the moment of giving birth. This made the pain bearable because I was getting successful.*^*L, SP, PVD*^.

*The contractions were intense*,* almost like stomachache*,* but they were not painful suffering at all. It never crossed my mind to request medication. It was something I had to handle on my own. I knew that medical interventions would complicate my childbirth.*^*L, AU, CS*^.

The second group consisted of mothers who, finding the pain unbearable, opted for medical interventions such as epidurals or even caesarean sections. In their view, labor pain was not only unhelpful to the progress of childbirth but also dangerous. They believed that, in the present era, vaginal birth was unnecessary when a safe method such as caesarean section was available. Their perspective was shaped by the negative vaginal birth experiences of their friends and acquaintances as well. These negative attitudes, preconceptions, and feelings of insecurity about vaginal birth might undermine the self-confidence of mothers in coping with pain and even affect their relationship with healthcare staff.

*Why should I endure all this pain when I can easily have surgery? Why should I jeopardize my own health and my baby’s? These were the questions I asked the medical team with a scream.*^*H, IN, CS*^.

*Most of my friends have had* caesarean *sections and been very satisfied. However*,* those who had vaginal birth told me it was unbearable. I could not endure so much pain*,* so before my labor*,* I requested a* caesarean *section*,* but my doctor rejected [my request]. Throughout the entire labor*,* I kept begging my husband: “tell them to do a caesarean section. Nobody would listen to me*,* and I could not understand them.*^*L, IN, CS*^.

*I felt so bad that I thought I might die. The pain was excruciating*,* and I was only dilated to 4 centimeters. I was sure I would not possibly be dilated to 10 centimeters. I was screaming and praying to God to let my baby survive.*^*L, AU, CS*^.

Based on their prior experiences of pain, some mothers perceived labor pain as threatening as other types of pain. The intense fear eroded their self-confidence in pain management. Concepts such as having a low pain tolerance indicated the mother’s preconceived notions about labor pain, directly and indirectly reinforcing the belief that she could not manage the pain. Some mothers were not even willing to attend prenatal classes due to their intense fear of childbirth. They hoped for epidurals or caesarean sections. This lack of knowledge about the stages of labor ultimately leaded to personal inefficiency in pain management.

*My pain tolerance is low. I absolutely cannot tolerate pain. When they told me to push*,* I thought something in my stomach might tear. I could not focus properly on the medical team’s instructions.*^*L, IN, CS*^.

… *I had a fear of pain from the very beginning of my pregnancy… The contractions were so painful that I found myself screaming and asking for an epidural.*^*H, IN, CS*^.

#### External environment

While it is true that all human understandings of events are internal, in this article, the term “external environment” was used, with the consensus of the authors, to distinguish external events from internal events. This research showed that the birthing environment influenced mother’s perception of pain and her ability to manage it. In other words, the external world, including spaces and people, could affect the mother’s perception of pain, either amplifying or alleviating its intensity. Based on the mothers’ images of the outside, the external environment included three subfactors: “spatial environment,” “social environment,” and “interactive environment”. This means that a mother’s positioning in the spatial environment, her interactions with her supportive ones, and even the interactions between individuals and the spaces could alter the intensity of her pain.

#### Spatial environment

The findings of this study indicated that the design of childbirth spaces, from the moment of admission to the final stage of childbirth, had a significant impact on mothers’ interpretation of pain. Being in spaces with positive architectural and physical stimuli, mothers felt a sense of security and capability. They experienced pain, but spatial safety stimulated a sense of security, enhanced their ability to manage pain, and conveyed the message to mothers that the space was at their disposal to have a successful childbirth.

*When I entered the labor ward*,* I was in pain. I had thought the ward was filled with mothers screaming*,* but it was quiet… There were no hospital gowns or white walls in sight… They even allowed me to wear the clothes I wanted… I still had a lot of pain*,* but I no longer felt negative… I felt like I was a guest at someone’s*,* and believed I could handle the pain.*^*H, SP, PVD*^.

*I was in pain and expected it to get worse. However*,* the birthing ball and a warm shower helped me a lot.*^*H, AU, NVD*^.

The physical environment of the childbirth space could indeed have an adverse effect, possibly amplifying the burden of stress and insecurity on mothers. With each negative stimulus present in the environment, they increasingly lost their ability to control the situation and sought to find relief from these conditions by requesting medication. The use of concepts such as blood and broken items indicated how mothers’ observations, starting from the moment they entered the hospital and stemming from inadequacies in physical design, had been effective in increasing pain severity and lowering their pain tolerance.

*When my husband and I entered the maternity triage*,* I witnessed someone bleeding. Seeing that blood made me stressed… When I was waiting for the elevator*,* various patients were in my sight… I got the feeling that I was one of them and started crying*,* begging my husband to take me back home.*^*L, AU, CS*^.

*When I entered the labor ward*,* many mothers were walking in the corridor. I did not want to see them. Most of them looked tired and stared at me. I was sitting in a wheelchair while waiting for my room to be prepared. I did not want to be there. In addition*,* the pain was overwhelming. Even recalling it now makes me nervous.*^*HS, IN, CS*^.

*The room was so small that there was no space to walk. There were no windows*,* and I felt like this room would be my grave.*^*L, IN, NVD*^.

In some cases, childbirth environments had been designed in a way that did not encourage mothers to pursue vaginal birth. Conversely, by subjecting mothers to invasive instruments, they prepared mothers for a caesarean section.

*I had practiced using nonpharmacological pain relief tools during pregnancy. However*,* in my room*,* there were no usable tools. There were numerous monitors and equipment that I did not even know what they were for*,* and I thought they were all meant to be used for my childbirth.*^*H, IN, CS*^.

*The ward was full of commotion*,* and the corridors were filled with stretchers and wheelchairs. I thought I had come to the wrong place. I asked the staff: “Is this a surgical department?*^*L, AU, CS*^.

#### Social environment

This research demonstrated that individuals surrounding the mother played a significant role in bolstering her resilience and self-confidence during labor. These individuals included healthcare staff, partners, midwives, and trusted relatives. By understanding the nature of childbirth pain and the mother’s personality, they could assist her in unbearable conditions by offering words of encouragement. By receiving comfort and respect, the mother could navigate the birthing process with strength.

*I was happy that my husband was by my side. He knew nothing about childbirth*,* and my pain did not lessen because of him. Nevertheless*,* his presence was reassuring. He would tell me that we had been together from the beginning and would stay together to the end.*^*L, SP, PVD*^.

*The nurses were very calm. When I complained about my pain and stress for the umpteenth time*,* they smiled and reassured me that everything was under control. They never treated me disrespectfully at all.*^*H, AU, NVD*^.

The presence of trained companions had a significant impact on encouraging mothers to cope with pain. They could assist mothers in managing pain by using nonpharmacological pain relief techniques.

*I was still dilated to 6 centimeters*,* and I felt like I could not go on. Nevertheless*,* my midwife told me everything had been going great thus far. She told me it was not too late*,* and I could rest. She started doing deep breathing exercises with me while holding my hand. That was where I decided to continue.*^*L, IN, NVD*^.

In this study, when the mother felt secure, without a sense of abandonment or loneliness, she was better equipped to cope with the pain. However, if she felt left alone to endure the pain, she might start to give up, and her labor might not progress effectively.

*When the nurses were examining me*,* there was a commotion outside my room. Everyone rushed out and left me alone without any response. I thought one of the mothers had died*,* and I was left there alone to die as well.*^*H, IN, CS*^.

*My husband and I did not want him to be present during childbirth. It is not common in my family. However*,* when I could not bear the pain*,* I personally asked him to be by my side. He came*,* and I gave birth half an hour later.*^*H, IN, NVD*^.

#### Interactive environment

The interactive environment proved effective when the mother could engage in mutual interactions with the physical space and the people. In fact, the physical and social environments were helpful when they provided a space that allowed the mother to interact with her surroundings to meet her needs. In other words, the interactive environment was where the mother’s positive or negative perception of the physical characteristics or relationship with people in that environment taked place. If the birthing environment did not allow the mother to have efficient interactions with objects and people, the functionality of the environment was essentially compromised.

*My husband was present throughout labor. However*,* during examinations*,* since all the medical staff were women*,* he felt embarrassed. He did not have a proper physical placement in the room. I was more concerned about him rather than focusing on my labor.*^*L, IN, NVD*^.

*I was in pain all night and felt suffocated. Breathing in the fresh air on the terrace somewhat relieved this feeling. However*,* when the sun rose*,* the light was too intense*,* causing stress. By closing and covering the window*,* I felt better again.*^*L, SP, PVD*^.

*My room had a beautiful view outside. However*,* I could not open the window to use fresh air. Maybe the outside air would not have helped me*,* but the fact that my request to open the window went unnoticed left me feeling tired and nervous.*

*I was still dilated to 4 centimeters. I felt fine and thought I was progressing well. Nevertheless*,* during the examination*,* the staff started talking as if something was amiss. When I asked*,* they said everything was fine. However*,* I did not believe them because they kept saying things I did not understand. Since that moment*,* I found it increasingly difficult to breathe.*^*L, IN, CS*^.

## Discussion

Using a mother-centered approach, this research examined the contextual factors affecting mothers’ perceptions of labor pain. The findings indicated that the average effect of external and internal environments determined whether mothers could control pain on their own or required medical intervention. The mother’s mentality, shaped by her lived experience in her social, cultural, and economic context, had a significant impact on the mother’s conception of the labor pain. Each mother had a unique understanding of pain, stemming from the deepest layers of her perception, which determined how she could cope with it. However, the aspects of the context where childbirth occurred appeared to be highly influential in shaping mothers’ perception of pain. In fact, the external world had an equivalent internal image based on each mother’s personal interpretation of it, which consequently affected the way she tended to deal with pain. Therefore, labor pain was a dynamic [12], mental, and qualitative concept that changed in response to external environmental conditions during the labor process and varied among individuals based on the life experiences of each mother.

The environmental strategies that influence pain based on individual interpretation are dimensions that have not been greatly explored in the literature. However, the effect of the internal environment on pain management correlated with the findings from other studies where internal factors affect mothers’ experience of labor [26, 46]. The data from this study suggested that the internal environment comprises two subthemes: “personal beliefs” and “self-confidence and lack of fear”. When pain was interpreted as purposeful and essential for the process of delivering a baby, it became associated with positive cognitions, and mothers with such attitudes were more likely to effectively manage the pain. Some mothers with profound religious beliefs derived solace by placing trust in a higher power or surrendering control during the childbirth process. This perspective provided a framework for understanding the meaning of labor, which helped mothers to find solace in the idea that their pain led to a positive outcome, preventing them from suffering. This was because it was the perceived suffering that affected the progression of labor, hindered the process, and led to less efficient or prolonged labor [11, 24, 47–50]. Other studies also show that labor pain is described as a form of spiritual transcendence by mothers [26, 51, 52] and husbands [53]. The results of this study showed that spirituality could be associated with a sense of community and social support. Moreover, participating in rituals and ceremonies related to childbirth might positively impacted the mother’s emotional well-being during labor. In this study, activities such as prayer and meditation during labor provided mothers with a calm and focused mindset and a sense of strength and resilience, helping them to endure the pain.

However, self-confidence and lack of fear may play a more significant role in labor management. This study demonstrated that when a mother felt confident in her ability to handle the challenges of labor, it could positively impact her emotional state and coping mechanisms. Education and preparation, birth plans, supportive environments, and positive affirmations could cultivate this sense. Alternatively, fear and anxiety could trigger the body’s stress response, leading to the release of stress hormones such as cortisol and adrenaline, intensifying pain perception and making it feel more challenging to manage. According to Henriksen, high levels of fear during childbirth are associated with increased pain perception [54], decreased control [50], and a higher likelihood of seeking pain relief interventions, such as epidurals or analgesics [54]. Dencker, Rouhe and Elvander also suggest that mothers lose their control when they are unprepared for complications [55–57], and Mirghafourvand considers the sense of power as the most important factor for positive labor experience [26]. Gaining knowledge through attending prenatal classes, where the labor process, pain management techniques, and potential challenges are taught, may empower mothers and partners, increasing their ability to use personal capacity to reduce perceived pain.

Based on the findings of this study, it is important to note that the internal environment could impact one’s experience of pain, but it may not solely determine it. The external environment also had a significant impact on a mother’s perceived labor pain, coping mechanisms and overall childbirth experience. Despite the fact that the process was the same across all cases, the context where a mother experiences labor reflected the perceived intensity and the qualitative characteristics of pain, determining her coping mechanisms [12]. According to Moseley and Arntz [58], a change in the context of a noxious stimulus can result in a change in the perceived intensity and unpleasantness of the pain experience. The findings of this study demonstrated that the external environment had 3 major subfactors: social, physical, and interactive environments.

In this study, the physical environment was highlighted as the most influential external factor contributing to mothers’ experience of labor pain and their ability to manage it effectively. Creating a supportive and comfortable environment was essential for fostering a positive birth experience and optimizing pain and stress management. The mothers who participated in this research declared that passing through hospital security, walking in corridors, facing patients, and noisy environments influenced their emotional state and, in some cases, affected their perception of pain and the birthing process. Pirdel also shows that a noisy and crowded environment influences the mother’s perception of pain [59]. The availability of advanced medical technology in hospitals transformed them into spaces for caesarean sections rather than home-like spaces for vaginal birth. As a result, the subjective reflection of the hospital environment for the mother was characterized by medical interventions such as spinal, epidural, and ultimately caesarean sections. According to MASS design studies, hospital design is a risk factor for C-section, and buildings help or hinder the processes of labor for mothers. Mothers are often subjected to intense treatment environments, leading them to embrace interventions such as epidurals or even C-Sect. [60]. Hodnett also states that hospital birth centers are associated with lower rates of medical interventions during labor and birth, as well as higher levels of satisfaction compared to conventional hospitals. In this study, some key factors related to the physical environment that potentially improve the management of perceived pain are as follows: privacy and comfort, lighting, noise level, temperature and ventilation, use of birthing aids, aromatherapy, supportive seating and positions, natural light and nature, positive distractions, wayfinding, and supportive facilities for partners. The findings of other studies emphasize the direct role of the physical environment in human social and emotional behavior [61], its importance in successful physiological vaginal birth [62–65] and alleviating labor pain through yoga or music [66].

This study confirmed the findings of previous studies, which indicate that a social environment with positive communication, compassionate caregivers, the presence of a partner, and a reassuring atmosphere can alleviate anxiety and stress for the mother. Feeling respected [46, 67] and confident [12], instead of merely relying on medical intervention, could positively influence the mother’s ability to cope with labor pain. Mothers participating in this study stated that the behavior and attitude of healthcare providers and empathetic individuals such as a partner, family member, or trained doulas, who listened to the mother’s needs and preferences, could enhance her sense of control and encouraged her to be strong and manage labor pain more effectively through emotional support, guidance, and reassurance. Other studies show that continuous labor support, as part of nonpharmacological approaches to pain management, reduces stress, which in turn decreases the frequency of obstetrical interventions [29], leading to a successful physiological vaginal birth [26, 68–71]. Since medical interventions contribute to the rising rate of caesarean Sect. [72]. Alternatively, feelings of being disrespected [73] or experiencing loneliness [12] contribute to a negative childbirth experience. The findings of this research on attitudes toward the partner’s presence contradicted those in the Western context [10, 46]. Based on the findings of this research, given the sociocultural context of Iran, many of the partners were reluctant to participate in the birthing process, and even the mothers agreed with their decision. However, when pain became overwhelmingly intense, mothers demanded the presence of their partners, and interestingly, the presence of their partners enhanced the mother’s ability to manage labor pain. This corroborated the findings of previous studies, which indicate that the presence of a trained husband can reduce the mother’s stress [24, 74, 75], and health professionals should address the unpleasantness of pain [24, 47].

The last subfactor of the external environment factor was the interactive environment, which served as an active participant in social relationships. This factor constantly changes and communicates with users [76]. In an interactive space, pragmatic or humanistic functions are accommodated to reconfigure itself in response to input from users and adapt its spatial performance to align with new emerging needs [77]. Within such an environment, the mother can have a sense of belonging to the space [78], as it provides a home-like space that satisfies her sense of control. This study demonstrated that an interactive environment, which helped to enforce productivity and improve mental and emotional states, could shape compelling experience and mothers’ perception of pain. In other words, communication with the external environment, such as control over lights, temperature, sounds, exits and entrances, and control over the process of childbirth could enhance her sense of empowerment and reduce feelings of helplessness. This sense of control played a role in improving pain management, helping to reduce the perception of pain. Other studies confirm these findings, which suggest that mothers’ participation in decision-making processes should be valued [79, 80] to give them a sense of control [79, 81] and belonging within the environment [79]. This in turn reduces pain intensity [82] and enhances the overall childbirth experience [83].

According to the findings of this study, the creation of an interactive space required engaging all the senses of the mother, including visual, auditory, olfactory, tactile, and even gustatory experiences. To achieve this, a multimodal design combining different modes of interaction, such as images, audio, video, touch, and movement, could be used. These findings supported Knox’s research, which suggests that the interactive environment triggers various senses, thereby stimulating further communication and influencing users’ behavior [84]. Within this environment, mothers could ask questions, their birth plans and preferences were respected, and compassionate care was provided, which involved active listening and partnership in decision-making. According to Knox, interaction facilitates a bidirectional dialog between space and users, where both parties have agency. This in turn enables real-time multiple loop conversation [85]. In fact, the built environment became interactive and adaptive through the integration of dialogs between mothers, supporters and spaces. Therefore, the interactive environment was more than a physical or social space that empowered the mother and positively influenced her sense of control over labor pain.

Therefore, an interactive environment may have the potential to ensure the proper functioning of the spatial and social aspects of the setting by integrating technology, design, and human engagement. Although the basis of an interactive environment lies in the physical layout and the human communication that occurs within it, ineffective interactions may compromise the positive impact of well-designed support systems.

Finally, it is important to note that in this study pain perception and management were highly subjective and individual experiences that could vary from one person to another. Each mother’s perception of labor pain was unique and could be affected by a combination of physical, emotional, social, and psychological factors. It is suggested that healthcare providers communicate with the laboring mother and her support team to tailor the environment to her preferences. While there may be mothers who experience more severe pain for medical reasons, this research showed that creating an environment that reduced their stress and enhanced the possibility of receiving social support in an interactive atmosphere could lead to a more positive perception of labor pain and enhanced the ability to cope with it more effectively.

## Strengths and limitations

This study investigated the contextual aspects that may influence the subjective concepts associated with labor pain. The research methodology adopted in this study enabled authors to potentially seek a more complete understanding of this phenomenon and potentially promote holistic approaches to pain management, potentially encouraging noninvasive interventions and fostering a supportive birthing environment. The results of this research may provide insights for the Ministry of Health, architects, and healthcare practitioners, helping them potentially improve the environmental aspects of the maternity setting, potentially steer away from excessive medical interventions, and potentially reduce the rate of unnecessary caesarean sections.

Although mothers who participated in this study were selected from a diverse statistical population of two cities in Iran with different cultures, public and private hospitals, as well as differing pregnancy risk levels, the results of this experiment may not be representative of all mothers giving birth in Iran. To achieve more generalizable results, further extensive research is needed, encompassing a larger sample of Iranian mothers. Replicating this research in different regions, re-testing the survey, and comparing responses between mothers who had caesarean sections and those who experienced vaginal birth may lead to more reliable findings. Future research may also assess the significance of childbirth space indicators from the perspectives of both healthcare professionals and mothers, taking into account the economic implications.

## Conclusion

The purpose of this study was to understand the inherent subjectivity of labor pain and make it more accessible for interpretation, unraveling effective environmental strategies that aid in managing labor pain in Iran. Labor pain was an enigmatic and cognitive experience that could be improved through both internal and external environments.

In conclusion, it is hoped that the findings of this article may be utilized to enhance and reform the clinical conditions of maternity care units, ultimately helping mothers in pain management. These environmental approaches may lead to a reduction in medical interventions, which potentially results in a smaller number of unsuccessful physiological vaginal birth or dissatisfaction. These findings indicated that positive personal attitude and physical, social, and interactive components of the birthing environment could assist both mothers and healthcare practitioners in pain management and achieving a successful and satisfying childbirth experience.

## Data Availability

All data generated or analysed during this study are included in these published article: 10.61186/jhehp.9.2.91. 10.18502/jfrh.v17i3.13538. Other datasets used and/or analysed during the current study are available from the corresponding author on reasonable request.

## References

[1] Azami-Aghdash S, Ghojazadeh M, Dehdilani N, Mohammadi M. Prevalence and causes of cesarean section in Iran: systematic review and meta-analysis. Iran J Public Health. 2014;43(5):545. 26060756 PMC4449402

[2] Zamani-Alavijeh F, Araban M, Hassanzadeh A, Makhouli K. Contributing factors of pregnant women’s beliefs towards mode of delivery: a cross-sectional study from Iran. Matern Health Neonatol Perinatol. 2018;4:9. 10.1186/s40748-018-0077-1. [PubMed ID: 29744129]. [PubMed Central ID: PMC5930689]. 29744129 10.1186/s40748-018-0077-1 PMC5930689

[3] Safaei nezhad A, Rastegari L, Kharaghani R. Prevalence and predictors of cesarean section in zanjan-Iran during 2014–2016. Prev Care Nurs Midwifery J. 2017;7(3):47–55.

[4] World Health Organization. WHO statement on caesarean section rates. 2015 [https://www.who.int/reproductivehealth/publications/maternal_perinatal_health/cs-statement/en/]

[5] Darsareh F, Aghamolaei T, Rajaei M, Madani A. Determinants of caesarean birth on maternal demand in the Islamic Republic of Iran: a review. East Mediterr Health J. 2017;23(6):441–448. 10.26719/2017.23.6.441. PMID: 28836657. 10.26719/2017.23.6.441 28836657

[6] Johri M, Ng ESW, Bermudez-Tamayo C, Hoch JS, Ducruet T, Chaillet N. A cluster-randomized trial to reduce caesarean delivery rates in Quebec: cost-effectiveness analysis. BMC Med. 2017;15(1):96. 10.1186/s12916-017-0859-8. 28528578 10.1186/s12916-017-0859-8 PMC5439122

[7] Kingdon C, Downe S, Betran AP. Women’s and communities’ views of targeted educational interventions to reduce unnecessary caesarean section: a qualitative evidence synthesis. Reprod Health. 2018;15(1):130. 10.1186/s12978-018-0570-z. 30041661 10.1186/s12978-018-0570-z PMC6057083

[8] Latifnejad-Roudsari R, Zakerihamidi M, Merghati-Khoei E, Kazemnejad A. Cultural perceptions and preferences of Iranian women regarding cesarean delivery. Iran J Nurs Midwifery Res Feb. 2014;19(7 Suppl 1):S28–36. PMID: 25949249; PMCID: PMC4402990. PMC4402990 25949249

[9] Deys L, Wilson V, Meedya S. What are women’s experiences of immediate skin-to-skin contact at cesarean section birth? An integrative literature review. Midwifery;101. (2021). 10.1016/j.midw.2021.103063 34157585

[10] Ryding EL, Lukasse M, Kristjansdottir H, Steingrimsdottir T, Schei B. Pregnant women’s preference for cesarean section and subsequent mode of birth–a six-country cohort study. J Psychosom Obstet Gynecol. 2016;37(3):75–83. 10.1080/0167482X.2016.1181055 27269591

[11] Jenkinson. Bec & josey, natalie & Kruske, Sue. BirthSpace: An evidence-based guide to birth environment design. 10.13140/RG.2.1.3962.8964. (2014).

[12] Haines HM, Rubertsson C, Pallant JF, Hildingsson I. The influence of women’s fear, attitudes and beliefs of childbirth on mode and experience of birth. BMC Pregnancy Childbirth. 2012;12(1):1. 22727217 10.1186/1471-2393-12-55 PMC3449179

[13] Kountanis JA, Kirk R, Handelzalts JE, et al. The associations of subjective appraisal of birth pain and provider-patient communication with postpartum-onset PTSD. Arch Womens Ment Health. 2022;25:171–80. 10.1007/s00737-021-01154-z. 34250546 10.1007/s00737-021-01154-z

[14] Whitburn L, Jones L, Davey M-A, Small R. The meaning of labor pain: How the social environment and other contextual factors shape women’s experiences. BMC Pregnancy Childbirth. 2017;17. 10.1186/s12884-017-1343-3. 10.1186/s12884-017-1343-3 PMC5450354 28558667

[15] Melzack, R. and Katz, J. The Neuromatrix in Behavioral Medicine. In The Handbook of Behavioral Medicine, D.I. Mostofsky (Ed.). 10.1002/9781118453940.ch35 (2014).

[16] Whitburn LY, Jones LE, Davey M-A, Small R. Women’s experiences of labor pain and the role of the mind: an exploratory study. Midwifery. 2014;30(9):1029–35. 24820004 10.1016/j.midw.2014.04.005

[17] Ghanbari-Homayi S, Fardiazar Z, Meedya S, Mohammad-Alizadeh-Charandabi S, Asghari-Jafarabadi M, Mohammadi E, Mirghafourvand M. Predictors of traumatic birth experience among a group of Iranian primipara women: a cross sectional study. BMC Pregnancy Childbirth. 2019;19(1):1–9. 31117987 10.1186/s12884-019-2333-4 PMC6532129

[18] Rasouli M, Keramat A, Khosravi A, Mohabatpour Z. Prevalence and factors associated with episiotomy in Shahroud City, northeast of Iran. Int J Womens Health Reprod Sci. 2016;4(3):125–9.

[19] Jones L, Othman M, Dowswell T, Alfirevic Z, Gates S, Newburn M, et al. Pain management for women in labor: an overview of systematic reviews. Cochrane Database Syst Rev. 2012;3:CD009234. 10.1002/14651858.CD009234.pub2 PMC7132546 22419342

[20] Lindholm A, Hildingsson I. Women’s preferences and received pain relief in childbirth–a prospective longitudinal study in a northern region of Sweden. Sex Reprod Healthc. 2015;6(2):74–81. 25998874 10.1016/j.srhc.2014.10.001

[21] Anim-Somuah M, Smyth R, Jones L. Epidural versus nonepidural or no analgesia in labor. Cochrane Database Syst Rev. 2011;12:CD000331. 10.1002/14651858.CD000331.pub3 22161362

[22] Adams J, Frawley J, Steel A, Broom A, Sibbritt D. Use of pharmacological and nonpharmacological labor pain management techniques and their relationship to maternal and infant birth outcomes: examination of a nationally representative sample of 1,835 pregnant women. Midwifery. 2015;31:458–63. 25649472 10.1016/j.midw.2014.12.012

[23] Chaillet, Nils & Belaid, Loubna & Crochetière, Chantal & Roy, Louise & Gagné, Guy-Paul& Moutquin, Jean-Marie & Rossignol, Michel & Dugas, Marylène & Wassef, Maggy & Bonapace,Julie. Nonpharmacologic Approaches for Pain Management During Labor Compared with Usual Care: A Meta-Analysis. Birth. 41. 10.1111/birt.12103. (2014) 10.1111/birt.12103 24761801

[24] Julie Bonapace G-P, Gagné N, Chaillet R, Gagnon E, Hébert S, Buckley. 355-Physiologic basis of Pain in Labor and Delivery: an evidence-based Approach to its management, Journal of Obstetrics and Gynecology Canada, 40, issue 2, Pages 227–45, ISSN 1701–2163, 10.1016/j.jogc.2017.08.003. (2018). 10.1016/j.jogc.2017.08.003 29447711

[25] Desta M, Mekonnen S. Labor pain control and associated factors among women who gave birth at Leku primary hospital, southern Ethiopia. BMC Res Notes. 2019;12:619. 10.1186/s13104-019-4645-x. 31547839 10.1186/s13104-019-4645-x PMC6757368

[26] Mirghafourvand M, Meedya S, Mohammadi E, Mohammad-Alizadeh S, Jafarabadi, Mohammad, Ghanbari. Solmaz. Iranian women’s perception on the determinants of birth experience: a qualitative study. BMC Pregnancy Childbirth. 2022. 10.1186/s12884-022-05078-z. 36199065 10.1186/s12884-022-05078-z PMC9535943

[27] World Health Organization. WHO recommendations on intrapartum care for a positive childbirth experience. (2018). 30070803

[28] Wideman TH, Edwards RR, Walton DM, Martel MO, Hudon A, Seminowicz DA. The Multimodal Assessment Model of Pain: a Novel Framework for further integrating the Subjective Pain Experience within Research and Practice. Clin J Pain Mar;35(3):212–21. 10.1097/AJP.0000000000000670. PMID: 30444733; PMCID: PMC6382036. (2019). 10.1097/AJP.0000000000000670 PMC6382036 30444733

[29] Melzack R, Katz J, Pain. Wires Cogn Sci. 2013;4:1–15. 10.1002/wcs.1201 26304172

[30] Avila LA. Pain beyond biology. Pain. 2013;154:2569–76. 10.1016/j.pain.2013.07.003 23850424

[31] Smith JA, Osborn M. (2008) Interpretative Phenemenological Analysis. In: Smith, J.A., Ed., *Qualitative Psychology*: *A Practical Guide to Research Methods*, Sage, London, 53–80. 10.1002/9780470776278.ch10

[32] Whitburn LY. Labor pain: from the physical brain to the conscious mind. J Psychosom Obstet Gynecol. 2013;34(3):139–43. 10.3109/0167482X.2013.829033 23952172

[33] Smith JA, Osborn M. Interpretative phenomenological analysis as a useful methodology for research on the lived experience of pain. Br J Pain. 2015;9(1):41–2. 10.1177/2049463714541642. 26516556 10.1177/2049463714541642 PMC4616994

[34] Smith JA. Qualitative psychology: a practical guide to research methods. 3rd ed. London: SAGE; 2015.

[35] Smith JA, Osborn M. Interpretative Phenomenological Analysis. In: Breakwell GM, editor. Doing Social Psychology Research. Malden: Blackwell Publishing; 2004. pp. 229–54.

[36] Smith JA, Dunworth F. Qualitative methodology. In: Valsiner J, editor. Handbook of Development psychology. London: Sage; 2003. pp. 603–21.

[37] Vella J. In pursuit of credibility: evaluating the divergence between member-checking and hermeneutic phenomenology. Res Social Administrative Pharm. 2024;20. 10.1016/j.sapharm.2024.04.001. 10.1016/j.sapharm.2024.04.001 38575497

[38] Gunawan J. Ensuring Trustworthiness in Qualitative Research. Belitung Nurs J. 2015;1(1):10–1. 10.33546/bnj.4.

[39] Monaro S, Gullick J, West S. Qualitative Data Analysis for Health Research: a step-by-step example of Phenomenological Interpretation. Qualitative Rep. 2022;27(4):1040–57.

[40] Birt L, Scott S, Cavers D, Campbell, Christine, Walter, Fiona. Member checking: a Tool to enhance trustworthiness or merely a nod to Validation? Qual Health Res. 2016;26. 10.1177/1049732316654870. 10.1177/1049732316654870 27340178

[41] Collier-Reed B, Ingerman Åke, Berglund A. Reflections on trustworthiness in phenomenographic research: recognising purpose, context and change in the process of research. Educ as Change. 2009;13:339–55. 10.1080/16823200903234901.

[42] Thomson G, Crowther S. (2022). Attuning to trustworthiness and final reflections. 10.4324/9781003081661-13

[43] Shenton A. Strategies for ensuring trustworthiness in qualitative Research projects. Educ Inform. 2004;22:63–75. 10.3233/EFI-2004-22201.

[44] Deljoo Ghamgosar F, Yazdanfar SA, Sahragard Monfared NS, Litkouhi S, Yari H, Honarbakhsh M. Effective Environmental Factors in Encouraging Iranian Mothers to Have Physiologic Birth through Facilitating it: An Exploratory Factor Analysis. jhehp. 2023; 9 (2):91–99, 10.61186/jhehp.9.2.91

[45] Deljoo Ghamgosar F, Yazdanfar SA, Nikkhah E, Yari H, Honarbakhsh M. Husbands’ perception of environmental characteristics during participation in physiologic delivery. J Family Reprod Health. 2023;17(3):151–64. 38716293 10.18502/jfrh.v17i3.13538 PMC11070737

[46] Cabrera EM, Mauricio D. Factors affecting the success of women’s entrepreneurship: a review of literature. Int J Gend Entrepreneurship. 2017;9(1):31–65. 10.1108/IJGE-01-2016-0001.

[47] Fenwick J, Toohill J, Creedy DK, Smith J, Gamble J. Sources, responses and moderators of childbirth fear in Australian women: a qualitative investigation, midwifery, 31, issue 1, Pages 239–46, ISSN 0266–6138, 10.1016/j.midw.2014.09.003. (2015). 10.1016/j.midw.2014.09.003 25440298

[48] Adams SS, Eberhard-Gran M, Eskild A. Fear of childbirth and duration of labor: a study of 2206 women with intended vaginal delivery. BJOG. Sep;119(10):1238-46. 10.1111/j.1471-0528.2012.03433.x. Epub 2012 Jun 27. PMID: 22734617 (2012). 10.1111/j.1471-0528.2012.03433.x 22734617

[49] Hodnett ED, Simmons-Tropea DA. The Labor Agentry Scale: psychometric properties of an instrument measuring control during childbirth. Res Nurs Health. Oct;10(5):301 – 10. 10.1002/nur.4770100503. PMID: 3671777. (1987). 10.1002/nur.4770100503 3671777

[50] Cheung W, Ip WY, Chan D. Maternal anxiety and feelings of control during labor: a study of Chinese first-time pregnant women. Midwifery Jun. 2007;23(2):123–30. 10.1016/j.midw.2006.05.001. Epub 2006 Oct 20. PMID: 17055624; PMCID: PMC7130936. 10.1016/j.midw.2006.05.001 PMC7130936 17055624

[51] Taghizdeh Z, Ebadi A, Dehghani M, Gharacheh M, Yadollahi P. A time for psycho-spiritual transcendence: the experiences of Iranian women of pain during childbirth, women and birth, 30, Issue 6, Pages 491–6, ISSN 1871–5192, 10.1016/j.wombi.2017.04.010. (2017). 10.1016/j.wombi.2017.04.010 28652017

[52] Moloney S. Dancing with the wind: a methodological approach to researching women’s spirituality around menstruation and birth. Int J Qual Methods. 2007;6(1):114–25.

[53] Bélanger-Lévesque MN, Dumas M, Blouin S, et al. That was intense! Spirituality during childbirth: a mixed-method comparative study of mothers’ and fathers’ experiences in a public hospital. BMC Pregnancy Childbirth. 2016;16:294. 10.1186/s12884-016-1072-z. 27716107 10.1186/s12884-016-1072-z PMC5045591

[54] Dencker A, Nilsson C, Begley C, Jangsten E, Mollberg M, Patel H, Wigert H, Hessman E, Sjöblom H. Carina Sparud-Lundin, Causes and outcomes in studies of fear of childbirth: a systematic review, women and birth, 32, issue 2, Pages 99–111, ISSN 1871–5192, 10.1016/j.wombi.2018.07.004. (2019). 10.1016/j.wombi.2018.07.004 30115515

[55] Lena Henriksen E, Grimsrud B, Schei M, Lukasse. Factors related to a negative birth experience – A mixed methods study, midwifery, 51, Pages 33–9, ISSN 0266–6138, 10.1016/j.midw.2017.05.004. (2017). 10.1016/j.midw.2017.05.004 28528179

[56] Rouhe H, Salmela-Aro K, Toivanen R, Tokola M, Halmesmäki E, Ryding EL, et al. Group psychoeducation with relaxation for severe fear of childbirth improves maternal adjustment and childbirth experience-a randomized controlled trial. J Psychosom Obstet Gynecol. 2015;36(1):1–9. 10.3109/0167482X.2014.980722 25417935

[57] Elvander C, Cnattingius S, Kjerulff KH. Birth experience in women with low, intermediate or high levels of fear: findings from the first baby study. Birth. 2013;40(4):289–96. 24344710 10.1111/birt.12065 PMC3868996

[58] Moseley GL, Arntz A. The context of a noxious stimulus affects the pain it evokes. Pain;133(1–3):64–71. (2007). 10.1016/j.pain.2007.03.002 17449180

[59] Manizheh P, Leila P. Perceived environmental stressors and pain perception during labor among primiparous and multiparous women. J Reprod Infertil Oct;10(3):217–23. PMID: 23926472; PMCID: PMC3719331. (2009). PMC3719331 23926472

[60] Ariadne, labs + MASS, Designing Capacity for High Value Healthcare: The Impact of Design on Clinical Care in Childbirth. (2017).

[61] Hammond A, Foureur M, Homer CSE, Davis D. Space, place and the midwife : exploring the relationship between the birth environment, neurobiology and midwifery practice. Women Birth. 2013. 10.1016/j.wombi.2013.09.001. 24139678 10.1016/j.wombi.2013.09.001

[62] Setola N, Naldi E, Cocina GG, Eide LB, Iannuzzi L, Daly D. The impact of the physical environment on Intrapartum Maternity Care: identification of eight crucial building spaces. HERD: Health Environ Res Des J. 2019;12(4):67–98. 10.1177/1937586719826058. 10.1177/1937586719826058 30767614

[63] Nielsen JH, Overgaard C. Healing architecture and Snoezelen in delivery room design: a qualitative study of women’s birth experiences and patient-centeredness of care. BMC Pregnancy Childbirth. 2020;20:283. 10.1186/s12884-020-02983-z. 32393297 10.1186/s12884-020-02983-z PMC7216688

[64] Aune I, Torvik HM. Promoting a normal birth and a positive birth experience — Norwegian women’ s perspectives. Midwifery. 10.1016/j.midw.2015.03.016. (2015). 10.1016/j.midw.2015.03.016 25907004

[65] Rados M, Mészáros J. A támogató környezet szerepe a szülési stressz kezelésében [The role of environmental factors in managing labor stress]. Orv Hetil. Jul;158(29):1149–1156. Hungarian. 10.1556/650.2017.30797. PMID: 28714330 (2017). 10.1556/650.2017.30797 28714330

[66] Smith CA, Levett KM, Collins CT, Armour M, Dahlen HG, Suganuma M. Relaxation techniques for pain management in labor. Cochrane Database of Systematic Reviews 2018, Issue 3. Art. No.: CD009514. DOI:, Karlström A, Nystedt A, Hildingsson I. June. The meaning of a very positive birth experience: focus groups discussions with women. *BMC Pregnancy Childbirth* 15, 251. 10.1186/s12884-015-0683-0 (2015). 10.1186/s12884-015-0683-0 PMC4600272 26453022

[67] Anteneh Asefa A, Semaan T, Delvaux E, Huysmans A, Galle E, Sacks MA, Bohren A, Morgan M, Sadler S, Vedam L, Benova. The impact of COVID-19 on the provision of respectful maternity care: Findings from a global survey of health workers, Women and Birth, Volume 35, Issue 4, Pages 378–386, ISSN 1871–5192, 10.1016/j.wombi.2021.09.003. (2022). 10.1016/j.wombi.2021.09.003 PMC9179099 34531166

[68] Sigurdardottir VL, Gamble J, Gudmundsdottir B, Kristjansdottir H, Sveinsdottir H, Gottfredsdottir H. The predictive role of support in the birth experience: a longitudinal cohort study. Women Birth. 2017;30(6):450–9. 28478933 10.1016/j.wombi.2017.04.003

[69] Neerland CE, Skalisky AE. A Qualitative Study of US Women’s Perspectives on Confidence for Physiologic Birth in the Birth Center Model of Prenatal Care. JMWH;67(4):435–41. (2022). 10.1111/jmwh.13349 35246924

[70] İsbir GözdeGökçe1*, Serçekuş. Pinar2. The Effects of Intrapartum Supportive Care on Fear of Delivery and Labor Outcomes: A Single-Blind Randomized Controlled Trial. Journal of Nursing Research 25(2):p 112–119, April. | 10.1097/JNR.0000000000000129 (2017). 10.1097/JNR.0000000000000129 28277391

[71] Bohren MA, Hofmeyr GJ, Sakala C, Fukuzawa RK, Cuthbert A. Continuous support for women during childbirth, Cochrane Database of Systematic Reviews, 7, John Wiley & Sons, Ltd, 1465–1858, Pregnancy and Childbirth, 10.1002/14651858.CD003766.pub6 (2017). 10.1002/14651858.CD003766.pub6 PMC6483123 28681500

[72] Sabetghadam S, Keramat A, Goli S, Malary M, Rezaie Chamani S. Assessment of medicalization of pregnancy and childbirth in low-risk pregnancies: a cross-sectional study. Int J Community Based Nurs Midwifery. 2022;10(1):64–73. PMID: 35005042; PMCID: PMC8724730. 35005042 10.30476/IJCBNM.2021.90292.1686 PMC8724730

[73] Miyauchi A, Shishido E, Horiuchi S. Women’s experiences and perceptions of women-centered care and respectful care during facility-based childbirth: a meta‐synthesis. Jpn J Nurs Sci. 2022;7:e12475. 10.1111/jjns.12475 35133066

[74] Hoffmann L, Hilger N, Riolino E, et al. Partner support and relationship quality as potential resources for childbirth and the transition to parenthood. BMC Pregnancy Childbirth. 2023;23:435. 10.1186/s12884-023-05748-6. 37312055 10.1186/s12884-023-05748-6 PMC10261844

[75] Salehi A, Fahami F, Beigi M. The effect of presence of trained husbands beside their wives during childbirth on women’s anxiety. Iran J Nurs Midwifery Res Nov-Dec;21(6):611–5. 10.4103/1735-9066.197672. PMID: 28194202; PMCID: PMC5301069. (2016). 10.4103/1735-9066.197672 PMC5301069 28194202

[76] Knox K. Agency of Interactive Space in Social Relationship. 10.52842/conf.caadria.2.381 (2019).

[77] Oosterhuis K. Swarm Architecture II. Delft: TU Delft; 2006.

[78] Andeson S. Seductive Interaction Design: creating Playful, Fun, andEffective user experiences (voices that matter). Berkeley, CA: NewRiders; 2011.

[79] Lyn Ebert H, Bellchambers A, Ferguson J, Browne. Socially disadvantaged women’s views of barriers to feeling safe to engage in decision-making in maternity care, Women and Birth, Volume 27, Issue 2, Pages 132–137, ISSN 1871–5192, 10.1016/j.wombi.2013.11.003. (2014). 10.1016/j.wombi.2013.11.003 24355713

[80] Lucia Mazúchová S, Kelčíková L, Štofaníková J, Kopincová N, Malinovská. Marián Grendár, satisfaction of Slovak women with psychosocial aspects of care during childbirth, midwifery, 86, 102711, ISSN 0266–6138, 10.1016/j.midw.2020.102711. (2020). 10.1016/j.midw.2020.102711 32278231

[81] Downe S, Finlayson K, Oladapo OT, Bonet M, Gülmezoglu AM. What matters to women during childbirth: a systematic qualitative review. PLoS ONE. 2018;13(4):e0194906. 10.1371/journal.pone.0194906. 29664907 10.1371/journal.pone.0194906 PMC5903648

[82] Tinti C, Schmidt S, Businaro N. Pain and emotions reported after childbirth and recalled 6 months later: the role of controllability. J Psychosom Obstet Gynecol Jun. 2011;32(2):98–103. Epub 2011 Mar 2. PMID: 21366397. 10.3109/0167482X.2011.557756 21366397

[83] Stevens NR, Wallston KA, Hamilton NA. Perceived control and maternal satisfaction with childbirth: a measure development study. J Psychosom Obstet Gynecol. Mar;33(1):15–24. 10.3109/0167482X.2011.652996. PMID: 22304395, (2012). 10.3109/0167482X.2011.652996 22304395

[84] Boychenko KV, Boychenko IV, Kudryashova AY. Interactive Built Space as the New Means of Information Communication, IEEE International Scientific Conference Systems of Signal Synchronization Generating and Processing in Telecommunications (SYNCHROINFO), pp. 1–4, (2019).

[85] Boychenko K. Interactive Architecture: development and implementation into the built environment, European Journal of Technology and Design, vol. 15, March (2017).

